# Supplementary material for: Occurrence of foamy macrophages during the innate response of zebrafish to trypanosome infections
Source: eLife. 2021 Jun 11;10:e64520. doi: 10.7554/eLife.64520 (PMC8238505; doi:10.7554/eLife.64520)
Supplement: Transparent reporting form [file elife-64520-transrepform.pdf]

eLIFE
1st Floor
24 Hills Road
Cambridge CB2 1JP, UK
P 01223 855340
W elifesciences.org
T @elife

eLife's transparent reporting form

We encourage authors to provide detailed information *within their submission* to facilitate the interpretation and replication of experiments. Authors can upload supporting documentation to indicate the use of appropriate reporting guidelines for health-related research (see EQUATOR Network), life science research (see the BioSharing Information Resource), or the ARRIVE guidelines for reporting work involving animal research. Where applicable, authors should refer to any relevant reporting standards documents in this form.

If you have any questions, please consult our Journal Policies and/or contact us: editorial@elifesciences.org.

Sample-size estimation

- You should state whether an appropriate sample size was computed when the study was being designed
- You should state the statistical method of sample size computation and any required assumptions
- If no explicit power analysis was used, you should describe how you decided what sample (replicate) size (number) to use

Please outline where this information can be found within the submission (e.g., sections or figure legends), or explain why this information doesn't apply to your submission:

Our study mostly relies on *in vivo* imaging and description of the observed response with respect to cell morphology, localization and cytokine expression as indirectly measured by GFP-reporter expression; in some cases however, gene expression analysis or cell proliferation or total fluorescence was quantified as in figure 4-10. The data behind the graphs showed in each figure can be found in the accompanying Source data files.

For the gene expression analysis, as reference to determine the minimum number of samples required, we used previous work on trypanosome infection of common carp (Forlenza et al., 2009a, 2009b, 2008; Joerink et al., 2006; Piazzon et al., 2016). There, at least n=3 (preferably n=5) individuals were used to assess changes in gene expression in infected and non-infected individuals. In the current study therefore, we used n=3-6 pools of larvae at each time point to assess the progression of the infection. This information can be found in the methods section and in the legend of figure 2-3.

For analysis of total cell fluorescence, when entire larvae were imaged, then the larva was the experimental unit. For the experiment in figure 4, n=50-55 larvae per group were used. This allowed us to image a high number of individuals at each time point and at least n=5 at late time points in the high-infected group, were generally a higher mortality was observed. This information can be found in the legend of figure 4.

For analysis of total cell fluorescence in figure 5-6, the head or the trunk region of an individual were the experimental unit and at least n=4 preferably n=5 individuals were imaged in each experiment. This information can be found in the legend of figure 5-6.

Forlenza M, Magez S, Scharsack JP, Westphal A, Savelkoul HFJ, Wiegertjes GF. 2009a. Receptor-Mediated and Lectin-Like Activities of Carp (Cyprinus carpio) TNF-a. *J Immunol* **183**:5319–5332. doi:10.4049/jimmunol.0901780
Forlenza M, Nakao M, Wibowo I, Joerink M, Arts JAJ, Savelkoul HFJ, Wiegertjes GF. 2009b. Nitric oxide hinders antibody clearance from the surface of Trypanoplasma borreli and increases susceptibility to complement-mediated lysis. *Mol Immunol* **46**:3188–3197.

eLife Sciences Publications, Ltd is a limited liability non-profit non-stock corporation incorporated in the State of Delaware, USA, with company number 5030732, and is registered in the UK with company number FC030576 and branch number BR015634 at the address 1st Floor, 24 Hills Road, Cambridge CB2 1JP | August 2014

Forlenza M, Scharsack JP, Kachamakova NM, Taverne-Thiele AJ, Rombout JHWM, Wiegertjes GF. 2008. Differential contribution of neutrophilic granulocytes and macrophages to nitrosative stress in a host-parasite animal model. *Mol Immunol* **45**:3178–3189.

Joerink M, Forlenza M, Ribeiro CMS, de Vries BJ, Savelkoul HFJ, Wiegertjes GF. 2006. Differential macrophage polarisation during parasitic infections in common carp (Cyprinus carpio L.). *Fish Shellfish Immunol* **21**:561–571. doi:10.1016/j.fsi.2006.03.006

Piazzon MC, Wentzel AS, Wiegertjes GF, Forlenza M. 2016. Carp Il10a and Il10b exert identical biological activities in vitro, but are differentially regulated in vivo. *Dev Comp Immunol*. doi:10.1016/j.dci.2016.08.016

Replicates

- You should report how often each experiment was performed
- You should include a definition of biological versus technical replication
- The data obtained should be provided and sufficient information should be provided to indicate the number of independent biological and/or technical replicates
- If you encountered any outliers, you should describe how these were handled
- Criteria for exclusion/inclusion of data should be clearly stated
- High-throughput sequence data should be uploaded before submission, with a private link for reviewers provided (these are available from both GEO and ArrayExpress)

Please outline where this information can be found within the submission (e.g., sections or figure legends), or explain why this information doesn't apply to your submission:

information about how often each experiment has been performed and how many replicates were used or shown is always provided in the figure legend and can be found back in the accompanying source data of figure 2-10.

Supplementary videos also provide the separate stacks from which the maximum projections shown in figure 7 were generated; other supplementary videos provide the raw data of the image shown in the manuscript plus an additional biological replicate. All this information can then be found in the corresponding figure legends of the figures and supplementary videos.

Statistical reporting

- Statistical analysis methods should be described and justified
- Raw data should be presented in figures whenever informative to do so (typically when N per group is less than 10)
- For each experiment, you should identify the statistical tests used, exact values of N, definitions of center, methods of multiple test correction, and dispersion and precision measures (e.g., mean, median, SD, SEM, confidence intervals; and, for the major substantive results, a measure of effect size (e.g., Pearson's r, Cohen's d)
- Report exact p-values wherever possible alongside the summary statistics and 95% confidence intervals. These should be reported for all key questions and not only when the p-value is less than 0.05.

Please outline where this information can be found within the submission (e.g., sections or figure legends), or explain why this information doesn't apply to your submission:

the statistical analysis is described in the methodology section and details are provided in the figure legends on the relevant figures.

(For large datasets, or papers with a very large number of statistical tests, you may upload a single table file with tests, Ns, etc., with reference to sections in the manuscript.)

Group allocation

- Indicate how samples were allocated into experimental groups (in the case of clinical studies, please specify allocation to treatment method); if randomization was used, please also state if restricted randomization was applied
- Indicate if masking was used during group allocation, data collection and/or data analysis

Please outline where this information can be found within the submission (e.g., sections or figure legends), or explain why this information doesn't apply to your submission:

N/A

Additional data files ("source data")

- We encourage you to upload relevant additional data files, such as numerical data that are represented as a graph in a figure, or as a summary table
- Where provided, these should be in the most useful format, and they can be uploaded as "Source data" files linked to a main figure or table
- Include model definition files including the full list of parameters used
- Include code used for data analysis (e.g., R, MatLab)
- Avoid stating that data files are "available upon request"

Please indicate the figures or tables for which source data files have been provided:

Figure 2
Figure 4
Figure 5
Figure 6

eLife Sciences Publications, Ltd is a limited liability non-profit non-stock corporation incorporated in the State of Delaware, USA, with company number 5030732, and is registered in the UK with company number FC030576 and branch number BR015634 at the address 1st Floor, 24 Hills Road, Cambridge CB2 1JP | August 2014
